# Angiotensin-converting enzyme insertion/deletion polymorphism and susceptibility to Kawasaki disease: a meta-analysis

**DOI:** 10.4314/ahs.v17i4.6

**Published:** 2017-12

**Authors:** Yan Pan, Hongzhu Lu

**Affiliations:** 1 Department of Pediatrics, the First Affiliated Hospital of Yangtze University, Jingzhou, Hubei Province, China; 2 Medical College of Yangtze University, Jingzhou, Hubei Province, China

**Keywords:** ACE, I/D polymorphism, Kawasaki disease

## Abstract

**Background:**

The angiotensin-converting enzyme (ACE) I/D polymorphism has been reported to be associated with Kawasaki disease (KD), but studies to date present conflicting results.

**Objectives:**

The aim of this study is to derive a more precise estimation of the association between the ACE I/D polymorphism and KD risk.

**Methods:**

PubMed, EMBASE, CNKI and Wangfang databases were retrievaled to identify for relevant studies from inception to May 2017. Pooled odds ratios (OR) with 95% confidence intervals (CI) were calculated using Stata 12.0 software.

**Results:**

A total of 6 case-control studies comprising 634 patients and 458 controls were included in the meta-analysis, and we found a significant association between the ACE I/D polymorphism and KD risk (D vs I:OR = 0.81, 95%CI = 0.31–2.11;DD vs II: OR = 1.03, 95%CI = 0.42–2.54; DI vs II: OR = 1.44, 95%CI = 1.09–1.90; dominant model: OR = 1.43, 95%CI = 1.11–1.85; recessive model: OR = 1.21, 95%CI = 0.44–3.29 ). When stratified by sample size>200, this polymorphism is associated with an increased the risk of KD.

**Conclusion:**

The I/D polymorphism in the ACE gene may be associated with susceptibility to KD.

## Introduction

Kawasaki disease (KD) is an self-limited vasculitis that mainly affects young children^1^. Although KD was first described in 1967^2^, its etiology is still not fully understood. The clinical manifestations of KD include persistent fever, non-purulent conjunctivitis, diffuse mucosal inflammation, polymorphous skin rashes, indurative angioedema of the hands and feet, and non-suppurative cervical lymphadenopathy^3^. In about 20% of patients vasculitis will lead to coronary artery lesions as detected by echocardiography, showing this to be the principal cause of acquired heart disease of children^4^. Recent studies suggest that gene polymorphisms maybe associated with KD, such as the FCGR2A gene rs1801274 polymorphism^5^.

The renin-angiotensin system (RAS) has been implicated in modulating blood pressure and homeostasis of the cardiovascular system^6^. Angiotensin-converting enzyme (ACE) is an crucial circulating enzyme of the RAS. It catalyzes the conversion of angiotensin I to angiotensin II and mediates bradykinin degradation^7^. In addition, angiotensin II is a potent pro-inflammatory modulator that augments and perpetuates immune responses. The human ACE gene is located on chromosome 17q23 and a large number of polymorphisms have been identified. One intron 16 insertion/deletion (I/D, rs4646994) polymorphism of this gene is characterized by the presence or absence of a 287bp Alu repetitive sequence^9^. Homozygotes for the D allele have the highest plasma ACE levels, heterozygotes (ID) have intermediate levels, and homozygotes for the I allele have the lowest levels^10^.

Many studies have investigated the relationship between ACE I/D polymorphism and KD. The inconsistency of these results may have resulted from inadequate statistical power owing to small sample size and eco-geographical differences. Meta-analysis may overcome these limitations of individual research^11^. We performed this meta-analysis to arrive at a more accurate estimation of the association of ACE I/D polymorphism with KD risk.

## Materials and methods

### Literature search strategy

Computer searches of PubMed, EMBASE, CNKI and Wangfang databases were performed via the following key words: “ACE gene”, “Kawasaki disease/KD”, “I/D”, “single nucleotide polymorphism” and “genetic polymorphism”. Only human studies were selected. Additional articles were identified by a manual searching of the references of in the related original studies.

### Study selection

Articles included in the meta-analysis met the following inclusion criteria:1) relevant case-control studies of KD cases and healthy controls; 2) articles on the relation of the ACE I/D polymorphism and susceptibility to KD and 3) studies that included sufficient genotype information for extraction. Exclusion criteria were as follows: 1) not case-control studies; 2) case reports, reviews, or meta-analysis; 3) studies that were based on incomplete raw data.

### Data extraction

The collected data included the first author's surname, publication date, country of origin, ethnicity, the number of cases and controls, the genotype frequency of ACE I/D polymorphism and deviation from Hardy-Weinberg Equilibrium (HWE) of the control group.

## Statistical analysis

Fisher's exact test was used to test HWE for distributions of genotypes among controls. The strength of the correlation between ACE I/D polymorphism and susceptibility to KD was estimated by odds ratio (OR) and 95% confidence interval (95%CI) as follows: D vs I, a homozygote comparison (DD vs II), a heterozygote comparison (DI vs II), a dominant model (DD+DI vs II) and a recessive model (II+DI vs DD) between groups. The heterogeneity among these articles was checked via the I2 test. When I2 > 50% indicated heterogeneity across studies, the random effects model was used, otherwise the fixed effects model was performed. The sensitivity analysis was performed by used via omitting each individual article, and an individual article was suspected of excessive sensitivity if the point estimate of its omitted analysis was outside the 95% CI of the pooled analysis. To assess the potential publication bias, Begg's and Egger's tests were performed. All statistical tests were performed with STATA (version 12.0; Stata Corporation, College Station, TX).

## Results

### Study characteristics

The database search yielded 137 publications, of which both of the reviewers considered 10 to be potentially eligible. We excluded 4 of the articles during the second phase of the inclusion process. The remaining 6 articles were included in the combined analysis^12^–^17^. A The flow chart summarizing the study selection process is shown in Figure 1. Included studies were all performed in China, Japan or Korea. All studies were in agreement with HWE except Shim et al, Wan et al and Xie et al^15^–^17^. The principle characteristics of eligible studies are summarized in Table 1.

**Figure:**
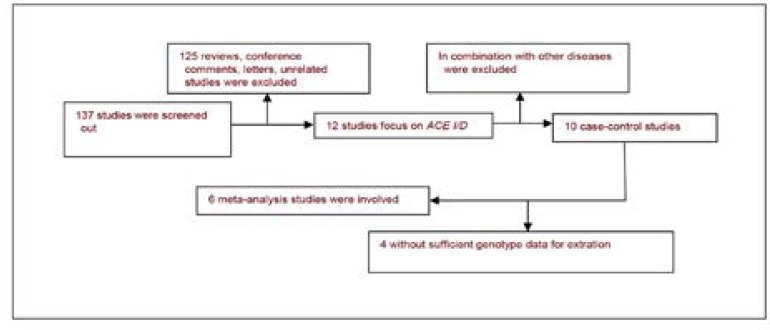


**Table 1:** Characteristics of the included studies for meta-analysis.

Study included	Year	Area	Race	Cases/Controls	Allele for cases		Allele for controls		Genotypes for cases			Genotypes for controls			HWE test

					D	I	D	I	DD	ID	II	DD	ID	II	
Wu	2004	China	Asian	107/107	83	131	88	126	6	71	30	18	52	37	0.97
Fuzakawa	2004	Japan	Asian	276/145	19	362	94	196	33	126	117	12	67	66	0.38
Liu	2005	China	Asian	28/35	44	12	36	34	18	6	4	9	19	7	0.60
Shim	2006	Korea	Asian	55/43	47	63	49	37	7	33	15	18	13	12	0.01
Wan	2006	China	Asian	138/98	167	109	78	118	60	47	31	24	30	44	0.00
Xie	2008	China	Asian	30/30	26	34	32	28	4	18	8	12	9	9	0.03

HWE: Hardy-Weinberg Equilibrium.

### Quantitative synthesis

**Figure 2:**
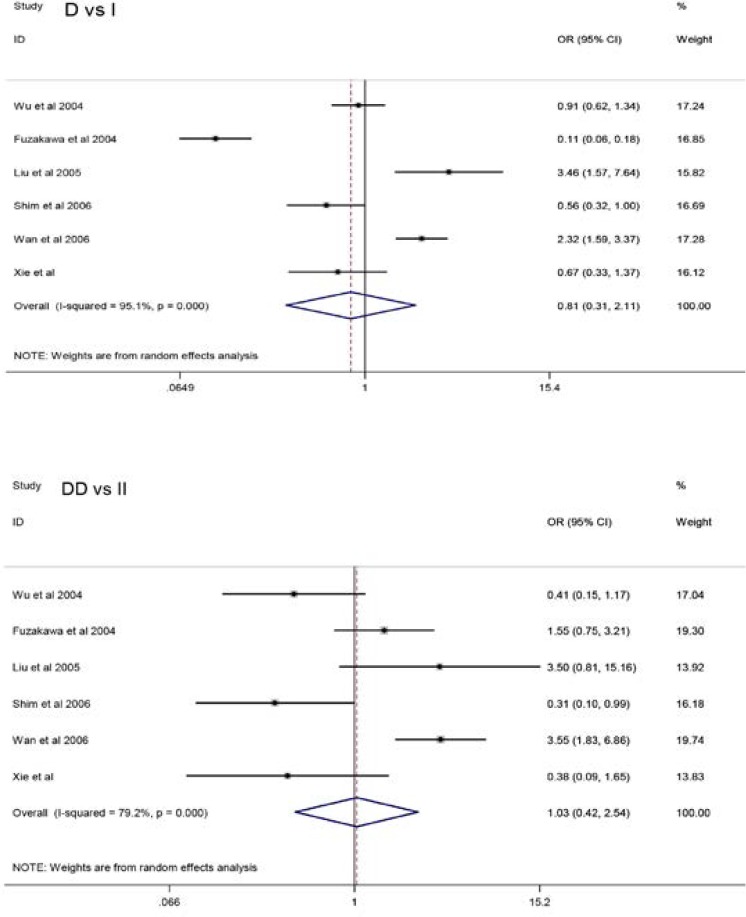

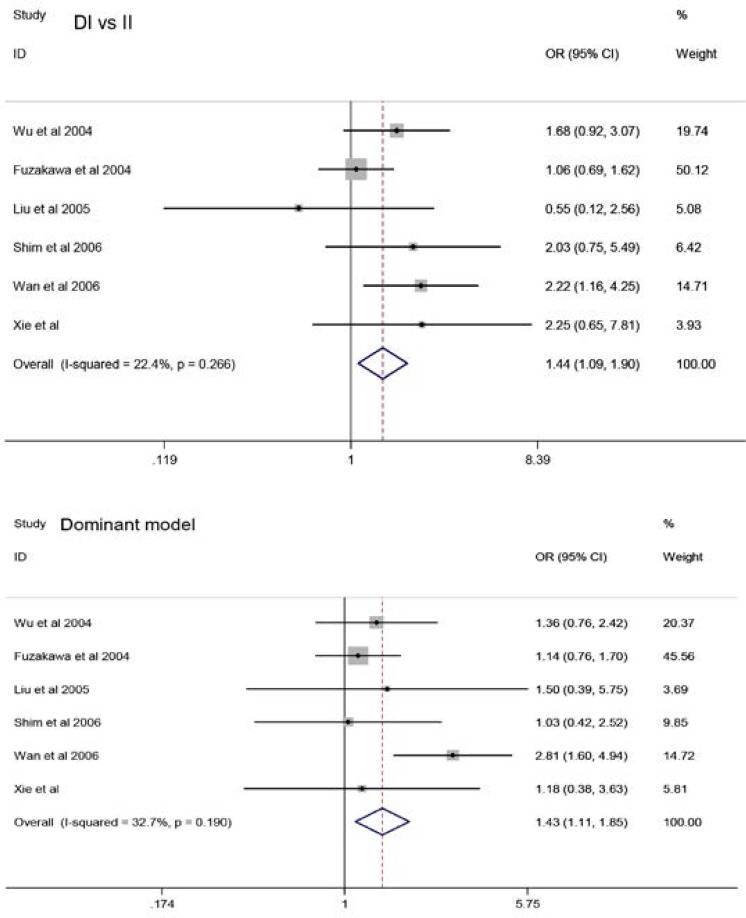

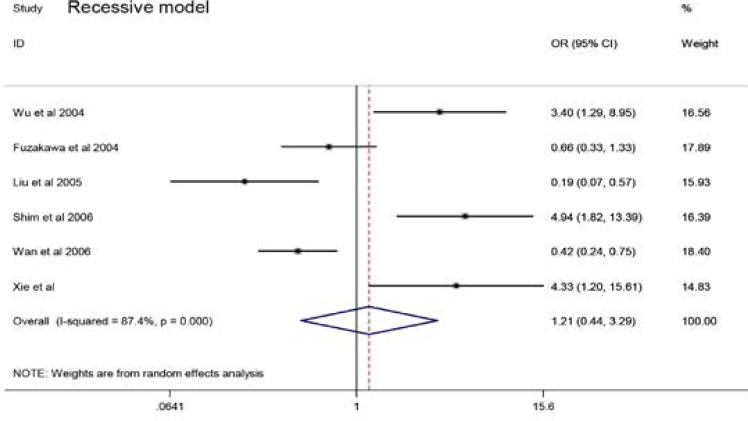
Forest plots for the association of ACE I/D polymorphism with risk of KD.

A summary of the meta-analysis findings of the association between ACE I/D polymorphism and KD risk is shown in Table 2 and Figure 2. Pooled analysis suggests that the ACE I/D polymorphism was significantly associated with KD(D vs I:OR = 0.81, 95%CI = 0.31–2.11;DD vs II: OR = 1.03, 95%CI = 0.42–2.54; DI vs II: OR = 1.44, 95%CI = 1.09–1.90; dominant model: OR = 1.43, 95%CI = 1.11–1.85; recessive model: OR = 1.21, 95%CI = 0.44–3.29 ). In sub-group analysis by sample size, the studies were divided into sample size >200 and sample size ≤200, and significant association was found between the ACE I/D polymorphism and KD risk in sample size>200 (D vs I:OR = 0.62, 95%CI = 0.12–3.11; DD vs II: OR = 1.40, 95%CI = 0.46–4.28; DI vs II: OR = 1.41, 95%CI = 1.04–1.91; dominant model: OR = 1.60, 95%CI = 0.93–2.74; recessive model: OR = 0.92, 95%CI = 0.31–2.74). However, when the analyses were restricted to small studies (n≤200 subjects), meta-analysis results showed no significant association. Moreover, when limiting the analysis to the study deviating from HWE, a significantly increased risk was observed (D vs I:OR = 0.98, 95%CI = 0.36–2.69; DD vs II: OR = 0.80, 95%CI = 0.13–4.84; DI vs II: OR = 2.18, 95%CI = 1.32–3.58; dominant model: OR = 1.66, 95%CI = 0.82–3.39; recessive model: OR = 1.98, 95%CI = 0.32–12.36).

**Table 2:** Summary of different comparative results.

	N	OR(95%CI)				
		
Variables		D vs I	DD vs II	DI vs II	Dominant model	Recessive mode
**Total**	6	0.81(0.31–2.11)	1.03(0.42–2.54)	1.44(1.09–1.90)	1.43(1.11–1.85)	1.21(0.44–3.29)
**Sample** **size**						
<200	3	0.62(0.12–3.11)	1.40(0.46–4.28)	1.41(1.04–1.91)	1.60(0.93–2.74)	0.92(0.31–2.74)
≤200	3	0.95(0.65–.38)	0.72(0.16–3.17)	1.60(0.80–3.18)	1.17(0.63–2.16)	1.59(0.19–13.43)
**HWE**						
Yes	3	0.69(0.12–4.04)	1.22(0.41–3.66)	1.19(0.85–1.67)	1.22(0.88–1.68)	0.77(0.18–3.30)
No	3	0.98(0.36–2.69)	0.80(0.13–4.84)	2.18(1.32–3.58)	1.66(0.82–3.39)	1.98(0.32–12.36)

N: number; CI: confidence interval; OR: odds ratio

**Figure 3:**
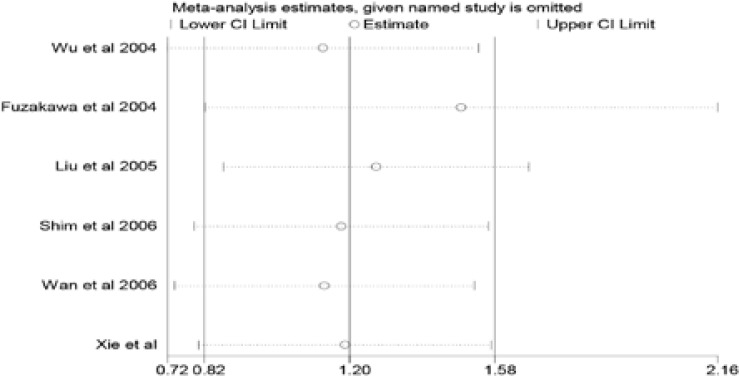
One-way sensitivity analysis of the pooled odds ratios and 95% confidence interval.

To evaluate the effect of a single article on the final result, we used a sensitivity analysis via removing one study at a time. Ultimately the pooled results hardly changed after removal of each study, suggesting that our results were robust (Figure 3).

### Publication bias

**Figure 4:**
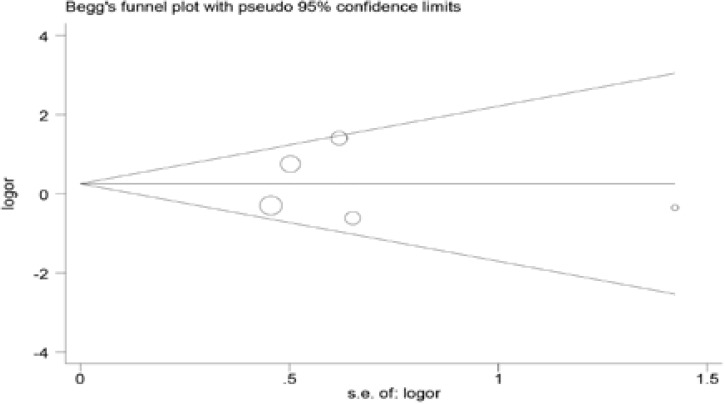
Begg's funnel plot test of publication bias.

**Figure 5:**
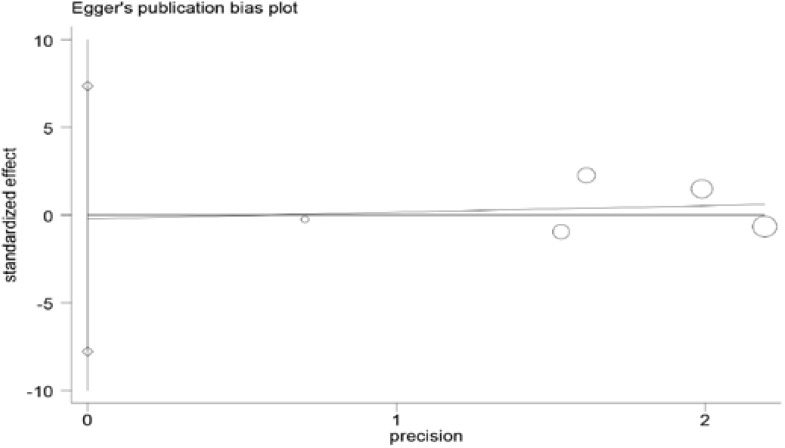
Egger's funnel plot test of publication bias.

Begg's and Egger's tests were used to assess the publication bias for ACE I/D polymorphism. The shape of the funnel plot did not reveal any evidence of obvious asymmetry, suggesting no evidence of publication bias for ACE I/D polymorphism (Figure 4 and Figure 5).

## Discussion

Although the morbidity is highest in Asians, KD is a major cause of acquired heart disease throughout the world^18^. After much investigation, the pathogenesis of KD is still not yet well understood. ACE not only increases vascular smooth muscle cell contraction, but also affects smooth muscle proliferation, monocyte adhesion, platelet adhesion, and aggregation^19^,^20^. To date, many studies have attempted to analyze the association between ACE I/D polymorphism and KD susceptibility, but the results have been are inconsistent. The aim of this meta-analysis was to investigate the possible association between ACE I/D polymorphism and KD risk based on relevant studies.

In this meta-analysis, we addressed the association between ACE I/D polymorphism and susceptibility to KD. Our results indicated that the ACE I/D polymorphism was significantly associated with the risk of KD. Nevertheless, considering that other potential factors might influence the final result, we conducted sub-group analysis. In a stratified analysis by sample size, pooled results showed significant association with sample size>200 but not with sample size≤200, suggesting that there was no small-study bias in this meta-analysis. The results of our study differ from a previous meta-analysis^21^. Our meta-analysis included six studies, and three recent studies by Liu et al, Wan et al and Xie et al were included in the present analysis. The previous meta-analysis performed by Lee et al suggested that the ACE I/D polymorphism is associated with several kinds of vasculitis (Behçet's disease and Henoch-Schonlein purpura), but not with KD. The author of the previous meta-analysis did not speculate as to the reasons for the disparate results. The difference may be due to small sample sizes.

Several limitations should be acknowledged of the current study. First, in the pooled analysis, we found that the ACE I/D polymorphism was significantly associated with the risk of KD in studies with PHWE<0.05. The data indicated that selection bias or genotyping error may have affected the merged results. Second, only the ACE I/D polymorphism was analyzed in this meta-analysis. Further analysis should clarify the association of other polymorphisms in the RAS genes, such as the AGT M235T and T174M polymorphisms. Third, we were unable to include unpublished studies, which might affect the publication bias. Additionally, there is a lack of information for the other population outside Asia. Therefore, the results of the current study are not comprehensive.

## Conclusion

Our pooled data showed a significant association between the ACE I/D polymorphism and the risk of KD. Due to the defect limitations of the included research, future large-scale investigations with appropriate design are required to confirm association.
